# Synaptic memory survives molecular turnover

**DOI:** 10.1073/pnas.2211572119

**Published:** 2022-10-10

**Authors:** Joel Lee, Xiumin Chen, Roger A. Nicoll

**Affiliations:** ^a^Department of Cellular and Molecular Pharmacology, University of California at San Francisco, San Francisco, CA 94158;; ^b^Department of Physiology, University of California at San Francisco, 600 16th Street Box 2140, GH-N272D San Francisco, CA94158

**Keywords:** CaMKII, synapse, memory

## Abstract

Activation of Ca^2+^/calmodulin-dependent kinase II (CaMKII) plays a critical role in long-term potentiation (LTP), a long accepted cellular model for learning and memory. However, how LTP and memories survive the turnover of synaptic proteins, particularly CaMKII, remains a mystery. Here, we take advantage of the finding that constitutive Ca^2+^-independent CaMKII activity, acquired prior to slice preparation, provides a lasting memory trace at synapses. In slice culture, this persistent CaMKII activity, in the absence of Ca^2+^ stimulation, remains stable over a 2-wk period, well beyond the turnover of CaMKII protein. We propose that the nascent CaMKII protein present at 2 wk acquired its activity from preexisting active CaMKII molecules, which transferred their activity to newly synthesized CaMKII molecules and thus maintain the memory in the face of protein turnover.

## Significance

How does the brain maintain memories that long outlast the proteins that encoded them? Francis Crick in 1984 proposed a model consisting of a multimeric protein whose subunits can phosphorylate each other and that naive unactive subunits can exchange into the active multimeric protein. An alternative model is that active holoenzymes can directly transfer activity to unactive holoenzymes, maintaining the integrity of the holoenzymes. Recent biochemical findings indicate that active Ca^2+^/calmodulin-dependent protein kinase II (CaMKII) can propagate activity to unactive CaMKII. Here, we demonstrate that hippocampal synapses contain a CaMKII memory trace acquired prior to slice culture preparation, which remains intact well after the complete turnover of CaMKII protein (2 weeks). We conclude that the synaptic memory (active CaMKII) can be transferred to naive newly synthesized CaMKII, thus sustaining the memory in the face of protein turnover.

A key question in the field of neuroscience is how memories are stored and how these memories outlast the lifetime of the molecules that encode them. For instance, long-term potentiation (LTP), a well-established cellular model for learning and memory can last at least 150 d (1), well beyond the life-time of most synaptic proteins, which generally have median half-life times of 2–5 d (2–5). This issue was initially addressed at a theoretical level with a two-step model (6, 7). The first step requires a multimeric protein with subunits that can phosphorylate each other. The second step requires that the protein undergoes subunit exchange such that naïve subunits can be exchanged into the active complex and be phosphorylated. Indeed, Ca^2+^/Calmodulin-dependent protein kinase II (CaMKII) satisfies the first step (8–10). CaMKII is a dodecamer in which Ca^2+^/CaM binding initiates both intra- and intersubunit phosphorylation within a holoenzyme. Remarkably it was shown that following a transient Ca^2+^ signal the kinase remained active (8, 10), a state referred to as autonomy. Thus, these results establish that CaMKII can convert a transient Ca^2+^ signal into one that can outlast the initiating stimulus. At about the same time, it was shown that CaMKII is required for the induction of LTP (11–13) and also mimics LTP (14–16). More recently, it has been found that CaMKII is required for maintaining LTP, at least during the first hour (17, 18), thus linking the first step of CaMKII autonomy to LTP.

Might CaMKII also satisfy the second step in which active CaMKII can transfer its activity to unactive CaMKII? Recent biochemical evidence suggests that this may be the case. Using total internal reflection fluorescence microscopy to track single molecules of CaMKII labeled with fluorescent markers, it was found that activation of CaMKII holoenzymes triggers colocalization of subunits between holoenzymes (19–22), including unactivated ones, resulting in the Ca^2+^-independent phosphorylation of new subunits (19). An additional/alternative mechanism for propagating CaMKII activity has recently been proposed (23). The authors show that in the presence of Ca^2+^/CaM, mixing wild-type holoenzymes with a kinase dead holoenzyme result in phosphorylation of the kinase dead CaMKII and this exchange still occurred when subunits were restrained to their parent holoenzyme by crosslinking the hub domains. Regardless of the specific mechanism, these studies establish that CaMKII can transfer its activity to naive CaMKII. Might this propagation of activity serve as a mechanism to maintain CaMKII activity and synaptic potentiation in the face of protein turnover? Interestingly, although the problem of memory stability and protein turnover was addressed in the mid-1980s, there has been no little physiological evidence that such a mechanism is operative.

CaMKII is known to turn over rapidly with a half-life of 2–4 d (2–4). Deleting CaMKII with CRISPR results in the complete loss of CaMKII protein, measured by Western blots and immunocytochemistry, within 2 wk (15). We have recently reported that synaptic transmission is maintained by Ca^2+^-independent constitutively active CaMKII, most likely reflecting a lasting memory trace induced by LTP prior to slice preparation (15, 18). Can constitutively active CaMKII remain active beyond 2 wk? To address this question, we have turned to hippocampal slice culture, which can be maintained for many weeks. In the presence of the NMDA receptor (NMDAR) antagonist APV and the Ca^2+^ channel antagonist nifedipine to prevent Ca^2+^ stimulation, we find that CaMKII activity once silenced remains inactive for two-weeks. This finding indicates that CaMKII cannot be reactivated under the resting conditions of our experiment. Second, we show that the constitutively active CaMKII remains unaltered throughout the 2-wk of slice culture. Given that CaMKII protein completely turns over within 2 wk, we propose that the CaMKII activity recorded at 2 wk in culture is generated by CaMKII that was not present at the beginning of the experiment, indicating the transfer of activity from old to new CaMKII protein.

## Results

**Fig. 1.**
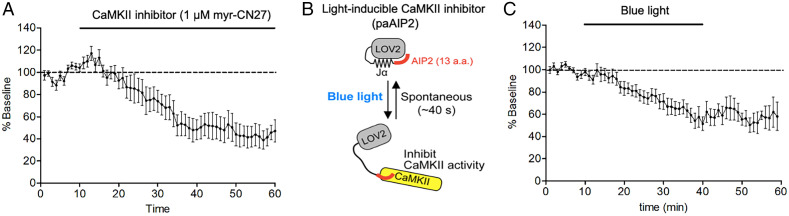
Inhibition of CaMKII depresses synaptic transmission. Slice cultures are bathed in APV and nifedipine to prevent Ca^2+^ entry into the cells. (*A*) Bath application of the membrane permeable CaMKII inhibitor myr-CN27 depresses synaptic transmission (*n* = 7). (*B*) Diagram of light-inducible CaMKII inhibitor (paAIP2) [reproduced from (24)]. (*C*) Exposure of cells transfected with paAIP2 to blue light depresses synaptic transmission (*n* = 11).

Our previous studies have shown that ∼50% of synaptic transmission is maintained by Ca^2+^-independent constitutive CaMKII activity, presumably acquired by prior LTP when the animal was alive (15, 18). In addition, we found that CaMKII protein, as measured by Western blotting and immunocytochemistry, is undetectable within 2 wk of CRISPR deletion of CaMKII (15). This loss of protein closely parallels the depression in synaptic transmission, indicating that these protein assays are an accurate reflection of the functional pool of CaMKII. Furthermore, the depression at 4 wk was no greater than at 2 wk (15). To address the impact of protein turnover on CaMKII activity, we have turned to hippocampal slice culture, which can be maintained for weeks in vitro. We first tested whether the CaMKII activity in slice culture is dependent on spontaneous Ca^2+^ transients by acutely applying the NMDAR antagonist APV (100 μM) and the Ca^2+^ channel antagonist nifedipine (20 μM). These inhibitors had no effect on the depression of synaptic transmission following CRISPR deletion of CaMKII in slice culture (*SI Appendix*, Fig. S1), indicating that the turnover of CaMKII was unaltered in the presence of these antagonists. We found that bath application of the membrane permeable CaMKII peptide inhibitor myr-CN27 (Fig. 1*A*) caused a roughly 50% depression of synaptic transmission, similar to that reported in the absence of inhibitors (15). We next transfected cells with paAIP2 (Fig. 1*B*), a novel light-inducible and reversible inhibitor of CaMKII (24). Light activation of paAIP2 also depressed synaptic transmission (Fig. 1*C*), indicating that, at least acutely, CaMKII activity is independent of Ca^2+^ influx through NMDARs or Ca^2+^ channels.

**Fig. 2.**
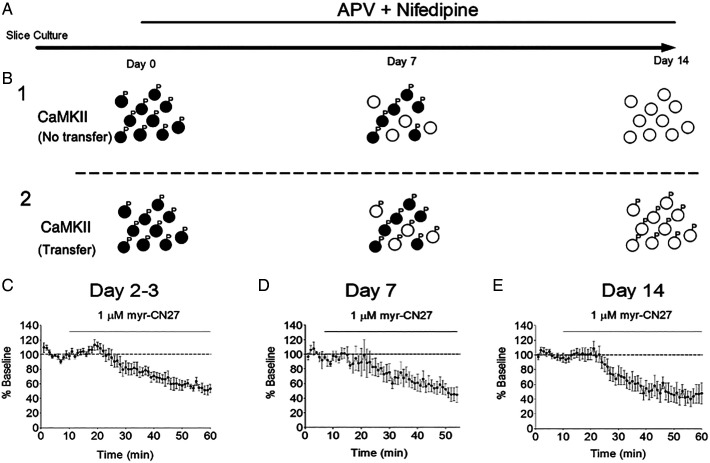
Constitutive CaMKII activity remains unaltered despite the complete turnover of CaMKII. (*A*) Timeline of the experiment. (*B*) Two scenarios are shown for the fate of CaMKII activity. (*B*_1_) CaMKII at the beginning of the experiment is illustrated as black circles with phosphates attached. CaMKII synthesized during the 2-wk period is illustrated with open circles. In this scenario the active CaMKII is degraded and activity is lost. (*B*_2_) In this scenario, the old CaMKII phosphorylates newly synthesized CaMKII. (*C*) After slice culture creation at P6–P8, slices were switched to slice culture media containing APV (100 μM) and nifedipine (20 μM). Recordings were carried out at various time points after the slice media was switched. All recording consisted of a baseline recording followed by changing the ACSF to one containing myr-CN27 (1 μM). Cells were recorded at various time points after solution change. (*C*) Recordings from days 2–3 after slice media was switched to APV and nifedipine (*n* = 7). (*D*) Recordings made at day 7 (*n* = 5). (*E*) Recording done on day 14 (*n* = 7). In all three cases, the myr-CN27 exhibited the same degree of depression.

Given that CaMKII protein completely turns over within 2 wk, we assayed constitutive CaMKII activity in slice culture over this time period (Fig. 2*A*). Is the constitutive activity lost as the active CaMKII is degraded (Fig. 2*B*1), or is the activity maintained by the transfer of the activity to newly synthesized CaMKII (Fig. 2*B*2)? To address this question, we assayed CaMKII activity over time with the CaMKII inhibitory peptide myr-CN27 (18) (see Fig. 1*A*). We found that CaMKII activity remained unaltered 2–3 d in culture (Fig. 2*C*), as well as 7 d (Fig. 2*D*) and, finally, 2 wk in culture (Fig. 2*E*). We conclude that, since the CaMKII has fully turned over by 2 wk, the CaMKII activity recorded at 2 wk has been acquired from preexisting active CaMKII before the onset of the APV and nifedipine treatment.

**Fig. 3.**
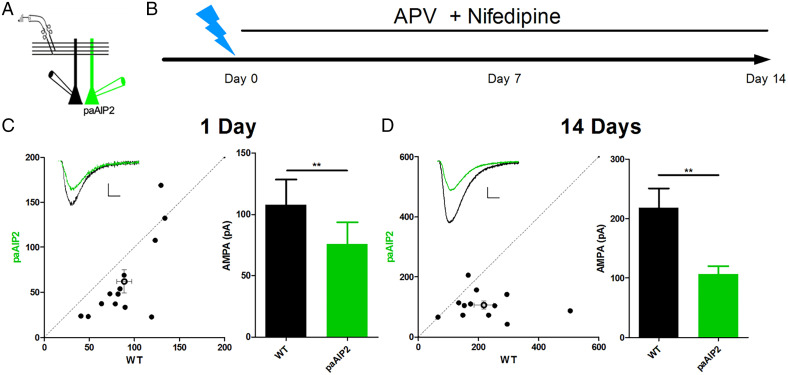
Constitutive CaMKII activity, once silenced, remains silent for 2 wk in hippocampal slice cultures. (*A*) A diagram depicting the paired, simultaneous recording scheme. (*B*) A timeline of the experiment. A day or two after preparing slice cultures from P6–P8 rats, gold bullets coated in plasmids for paAIP2 and a reporter pCAGG-mCh were used to transfect slices. At least 2 d after transfection, the slices were illuminated with a blue laser pulsing at 0.1 Hz for 1 s for 30 min (day 0). Slices were returned to the incubator and kept in slice culture media that contained APV (100 μM) and nifedipine (20 μM). Transfected cells and wild-type cells were then recorded and their AMPA currents compared. (*C*) Paired AMPA currents measured from 1 day after light illumination of the slice. The example trace shown has scale bars of 20 pA vertical and 10 ms horizontal. AMPA currents of transfected cells are significantly reduced (wild-type = 88.9 ± 8.3, Transfected = 62.1 ± 12.8, n =14 pairs). (*D*) Paired AMPA currents measured 14 d after light illumination of the slice. Example trace shown has scale bars of 50 pA vertical and 10 ms horizontal. AMPAR currents of transfected cells remain significantly reduced (wild-type = 218.1 ± 32.6, Transfected = 106 ± 12.9, *n* = 12 pairs). Analyzed with Wilcoxon signed rank test. (*C*) **P = 0.0052. (*D*) **P = 0.0049.

One concern with these findings is that, although we have prevented spontaneous Ca^2+^ entry through NMDARs and Ca^2+^ channels, we cannot be certain that intracellular Ca^2+^ over the 2-wk period might activate the newly synthesized CaMKII. To address this alternative, we turned to paAIP2 (see Fig. 1*B*) and paired recording from control and transfected cells (Fig. 3*A*). Blue light rapidly exposes the AIP2 peptide, which then returns to its closed inactive conformation in ∼40 s following light exposure (24). After slices were exposed to the blue light protocol (Fig. 3*B*), they were returned to the incubator and maintained in media containing APV and nifedipine. To monitor the depression over prolonged time periods, we compared the size of EPSCs in a transfected cell to that in a neighboring control cell (Fig. 3*A*). With this assay, CaMKII remained silent, not only for the first day following the exposure to blue light (Fig. 3*C*), but throughout the 2-wk period (Fig. 3*D*). These findings indicate that in the presence of APV and nifedipine, once silenced, CaMKII remains silent. There is no reactivation. Thus, we conclude that the CaMKII activity recorded at 2 wk (Fig. 2E) must have been transferred from preexisting active CaMKII. As an aside, the fact that CaMKII is not activated by non-NMDAR sources of Ca^2+^ during the 2 wk is important, because if this were to occur it would degrade the synapse specificity and Hebbian nature of LTP.

## Discussion

In the present study, we have addressed the long-standing problem of molecular storage in the face of protein turnover (6, 7). To address this problem, we have taken advantage of previous findings that synaptic transmission is maintained by the ongoing Ca^2+^/CaM-independent constitutive action of CaMKII (15, 17, 18). We have argued that this activity reflects an LTP memory trace acquired prior to slice preparation (18). However, it is important to emphasize that the origin of this activity is irrelevant for the question addressed in this study, which is whether this constitutive CaMKII activity remains following the complete turnover of CaMKII protein.

**Fig. 4.**
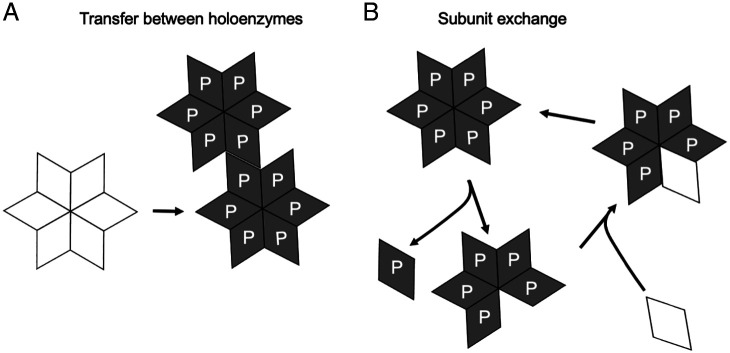
Models for the transfer of CaMKII phosphorylation to newly synthesized CaMKII. Only half of the 12-subunit structure of CaMKII is shown. (*A*) Shows a prion-like mechanism where phosphorylated holoenzymes phosphorylate unactive holoenzymes. The integrity of the holoenzymes remains intact. (*B*) Shows the exchange of phosphorylated subunits for unphosphorylated subunits. Modified from (9). It is important to note that the results presented in this study cannot distinguish between these two models. The “P” represents T286 phosphorylation. Although there is debate over the role of T286 phosphorylation in maintaining the action of CaMKII [e.g., (17, 51, 52)], our results are agnostic on the precise molecular basis for the constitutive CaMKII activity.

We find that in slices, cultured in the presence of the NMDAR antagonist APV and the Ca^2+^ channel blocker nifedipine, CaMKII, once silenced, remains silent for 2 wk. There is no reactivation under our conditions. However, we report that the constitutive action of CaMKII recorded shortly after slice culturing remains unaltered over this 2-wk period. There are two models that can explain the persistent action of CaMKII. First, an active holoenzyme could phosphorylate an unactive holoenzyme (Fig. 4*A*). While previous biochemical evidence has argued against this model (19, 25, 26), a recent study has proposed such a mechanism (23). This model runs the risk of uncontrolled runaway phosphorylation of unactive holoenzymes and the loss of synapse specificity, a feature of LTP. Presumably, the high levels of the phosphatase PP2A in the spine cytoplasm (27, 28), would limit the spread of active CaMKII. The second mechanism (Fig. 4*B*) involves the exchange of subunits in which a phosphorylated subunit in a holoenzyme is replaced by an unphosphorylated subunit, as originally postulated theoretically (6, 7, 9) and more recently biochemically (19, 20, 22). It is well accepted that CaMKII mRNA is localized and translated in dendrites, providing a local source for CaMKII replenishment (29–31). The attractiveness of the subunit exchange model is that it confines the propagation of the signal to those holoenzymes that were originally activated. It is important to emphasize that our results are agnostic regarding the mechanism by which CaMKII activity is propagated during protein turnover.

It is generally accepted that for CaMKII to enhance synaptic transmission, it must be bound to the C-tail of the GluN2B subunit of the NMDAR (15, 32, 33). This raises two additional intriguing questions. First, how does the binding of CaMKII to GluN2B interact with the propagation of CaMKII activity, be it either by subunit exchange or by interholoenzyme phosphorylation? Second, how does the CaMKII memory survive the turnover of NMDARs which is also complete within 2 wk (34)? Can activated CaMKII bound to one NMDAR bind to a new NMDAR? Answers to these questions will be of considerable interest.

A final consideration is the comparison of the present results to the sequence of events that are proposed to occur late after the induction of LTP. LTP is associated with a long-lasting increase in spine size (35, 36). This includes a late increase in the size of the PSD (37–40) and additionally a slow increase in the size of the presynaptic bouton (38, 39), resulting in the “matching” of pre- and postsynaptic structures following LTP. It is also proposed that protein synthesis is required for “late” LTP (37, 41, 42). One might expect that these late changes would be more stable, resisting reversal, and, perhaps, ultimately independent of CaMKII signaling. Our results indicate that at least for 2 wk, the magnitude and reversibility of CaMKII signaling remains unaltered. It is important to keep in mind that modeling mnemonic processes demonstrate that irreversible changes in synaptic weight are unstable and will quickly saturated (43–48). The interplay between constitutive CaMKII signaling and the associated synaptic structural changes remain to be explored.

## Materials and Methods

### Animals.

All the experimental procedures on animals were approved by the UCSF Animal Care and Use Committee. We usually used 5–7 animals to obtain a complete dataset.

### Experimental constructs and chemical agents.

The paAIP2 plasmid was obtained from Dr. Ryohei Yasuda (24). myr-CN27 was purchased from Calbiochem (catalog #208921).

### Slice culture and biolistic transfection.

For hippocampal slices, cultured 6 to 8-d-old rats were used (49). A helio gene gun with 1 μm DNA-coated gold particles (Bio-Rad) was used to biolistically transfect slices 1 d after sectioning. Slices were maintained at 34 °C and the medium was changed every 2 d. When experiments began, the slice media had APV and nifedipine added to it to concentrations of 100 μM and 20 μM.

### Photostimulation.

A 473-nm blue DPSS laser (Shanghai Laser & Optics Century, BL473T8-300FC) was used to deliver blue light pulses (0.1 Hz, 1 s, 20 mW/cm^2^). An optical patch cable connected to the optical fibers was used to deliver the blue light from the laser. A Master-8 (A.M.P. I) controlled the light pulses. When illuminating slices, they were superfused with artificial cerebrospinal fluid (ACSF) and either recorded from acutely or placed back into the slice culture dish and returned to the incubator.

### Electrophysiological recording.

Wild-type cells or fluorescent transfected pyramidal cells in CA1 region of hippocampus were recorded with patch electrodes (50). Dual recordings were made from control and transfected cells where noted. All recordings were made at 20–25 °C. Internal solution (in mM): 135 CsMeSO_4_, 8 NaCl, 10 Hepes, 5 QX314-Cl, 4 Mg-ATP, 0.3 Na-GTP, 0.3 EGTA, and 0.1 spermine. Osmolarity was adjusted to 290–295 mOm and pH was buffered at 7.3–7.4. External solution (mM): 119 NaCl, 2.5 KCl, 4 CaCl_2_, 4 MgCl_2_, 1 NaH_2_PO_4_, 26.2 NaHCO_3_, and 11 glucose, bubbled continuously with 95% O_2_/5% CO_2_. For most of the recordings, APV and nifedipine were also present in the external ACSF at concentrations of 100 μM and 20 μM, respectively. Synaptic currents were evoked every 10 s with bipolar stimulating electrodes placed in s. radiatum. To record EPSCs, picrotoxin (100 µM) was added to the external solution; for recording of AMPAR EPSCs, the cell membrane was held at −70 mV. A Multiclamp 700B amplifier (Axon Instruments), filtered at 2 kHz and digitized at 10 kHz, was used to record current responses. Cells with series resistance larger than 20 MOhm were excluded from analysis.

## Supplementary Material

Supplementary File

## Data Availability

All study data are included in the article and/or *SI Appendix*.
