# Supplementary material for: Synaptic memory survives molecular turnover
Source: Proc Natl Acad Sci U S A. 2022 Oct 10;119(42):e2211572119. doi: 10.1073/pnas.2211572119 (PMC9586278; doi:10.1073/pnas.2211572119)
Supplement: Supplementary File [file pnas.2211572119.sapp.pdf]

PNAS
www.pnas.org

Supplementary Information for

Synaptic memory survives molecular turnover

Joel Lee[1], Xiumin Chen[1] and Roger A. Nicoll[1,2]*

[1]Department of Cellular and Molecular Pharmacology and [2]Department of Physiology, University of California at San Francisco, San Francisco, CA

*Address all correspondence to: Roger Nicoll
Department of Cellular and Molecular Pharmacology
University of California at San Francisco
San Francisco, Ca
Email address: roger.nicoll@ucsf.edu
Phone: (415) 450-0265

This PDF file includes:

Supplementary text
Figures S1
SI References

Supplementary Information Text

Methods

Experimental Constructs and chemical agents

CaMKII DKO constructs had been described previously (1). APV and nifedipine were purchased from Sigma.

Slice culture and biolistic transfection

For hippocampal slices are made according to previous paper (1). A Helio Gene Gun with 1 μm DNA-coated gold particles (Bio-Rad) was used on slices for biolistically transfection, 2 day after slicing. Slices were maintained at 34°C and the medium was changed every 2 to 3 days. Once the slices had been made, its media had APV and nifedipine added to it to concentrations of 100 μM and 20 μM.

Fig. S1.

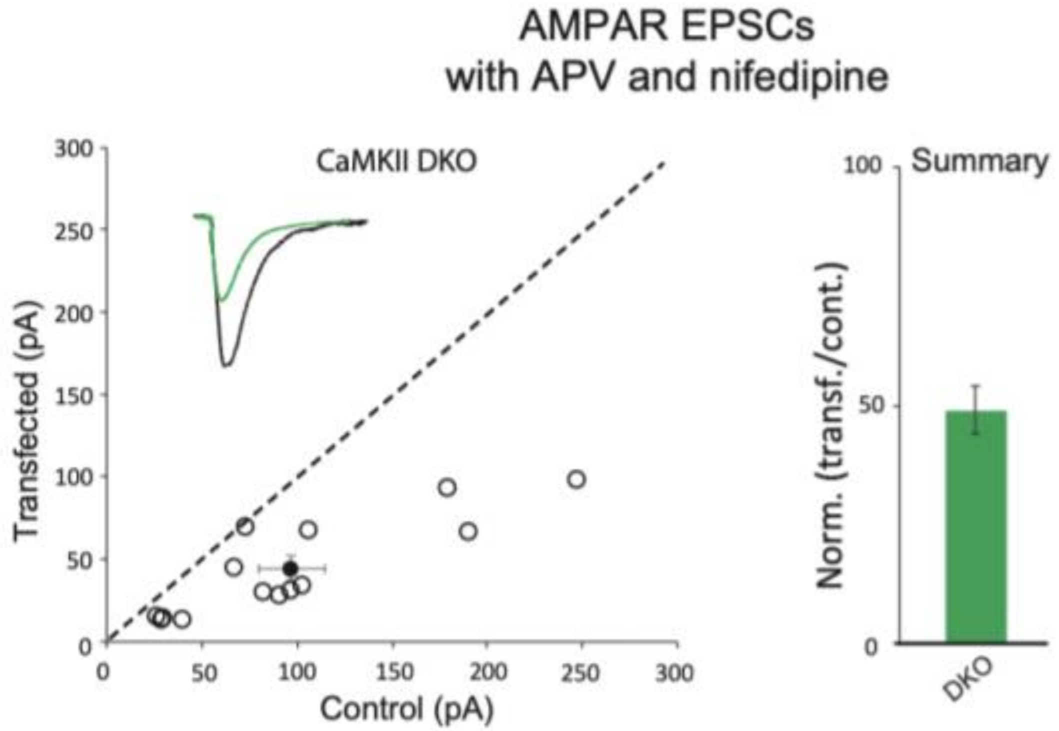

Figure S1. DKO of CaMKII still depresses synaptic transmission, while slice cultures are bathed in APV and nifedipine to prevent Ca^{2+} entry into the cells. Paired AMPA currents measured between CaMKII DKO and control cells. The example traces shown in green (CaMKII DKO cell) and black color (control cell). AMPA currents of transfected cells are significantly reduced. (WT = 97.1 ± 17, TF = 44.6 ± 8, n =14 pairs.)

References

1. S. Incontro *et al.*, The CaMKII/NMDA receptor complex controls hippocampal synaptic transmission by kinase-dependent and independent mechanisms. *Nat Commun* **9**, 2069 (2018).
